# An empirical analysis of the coupling and coordinated development of new urbanization and ecological welfare performance in China’s Chengdu–Chongqing economic circle

**DOI:** 10.1038/s41598-024-64141-1

**Published:** 2024-06-08

**Authors:** Jie Yang, Zhigang Li, Dong Zhang, Jialong Zhong

**Affiliations:** 1 College of Management Science, Chengdu University of Technology, Chengdu, 610059 The People’s Republic of China; 2 The Engineering & Technical College of Chengdu University of Technology, Leshan, 614000 The People’s Republic of China; 3 Research Center for Protection Policy of Key Ecological Functional Areas in the Upper Reaches of the Yangtze River, Chengdu, 610059 The People’s Republic of China

**Keywords:** Ecological welfare performance, New urbanization, Coupling coordination degree, GTWR model, Sustainability, Environmental sciences, Environmental social sciences

## Abstract

New urbanization (NU) and ecological welfare performance (EWP) play pivotal roles in achieving sustainable urban development, with both emphasizing social equity and environmental management. Exploring the coordinated relationship between EWP and NU is invaluable for understanding the symbiotic interplay between humans and nature. We constructed a framework to elucidate the coupling mechanism of EWP and NU from the perspective of systems theory. We quantified the levels of NU and EWP utilizing the entropy weighting method and the super-efficient SBM method, respectively. Furthermore, we assessed the degree of coupling coordination between the two using the coupling coordination degree model (CCDM). Spatial and temporal evolution analysis was conducted, and factors influencing the degree of coupling coordination between EWP and NU were explored through a spatial–temporal geographically-weighted regression model (GTWR). The results indicate: (1) During the study period, the average annual increase in EWP in the study area was 2.59%, with a narrowing relative gap between cities. Conversely, the average annual increase in the level of NU was 7.6%, with demographic and economic dimensions carrying the highest weights. (2) The type of coupling coordination between EWP and NU transitions from basic coordination to moderate coordination, with the development of EWP lagging behind that of NU. (3) City size demonstrates a positive yet diminishing trend on the coupling coordination level, with economic development exerting the greatest influence and exhibiting a "V" trend, while the impact of green technology innovation diminishes negatively. Additionally, regional disparities are significant, with city size exhibiting a negative impact in areas of high population density and low economic levels, and green technology innovation showing notable polarization characteristics in core cities. These findings serve as a foundation for fostering coordinated ecological development amid the rapid urbanization process of the Chengdu–Chongqing Economic Circle.

## Introduction

The interactive coupling and coordinated development between ecological environment and urbanization has become a key research area in the field of regional sustainable development^1,2^. The unprecedented scale and speed of urbanization in China provides a unique opportunity to study the rapid urbanization transition and its impact on the ecological environment^3,4^. Moreover, China's unique economic growth model, which combines elements of state guidance and market economy, provides a special context for exploring the complex relationship between economic development and ecological welfare^5,6^. The ability of the Chinese government in policy formulation and planning implementation, especially the strategic deployment embodied in the regional planning of the Chengdu–Chongqing Economic Zone, provides strong support for achieving the regional development goals. At the same time, China's deep cultural traditions and social values, especially in terms of environmental awareness and community engagement, provide a unique perspective for understanding the social dimension of urbanization^7,8^. In the face of environmental challenges, China's leading position in environmental technology and policy innovation provides valuable case studies on sustainable development^9^. The regional differences within China, especially the comparison between Chengdu–Chongqing economic circle and other regions, provide an opportunity to study the factors influencing different urbanization patterns and ecological welfare performance^10^. In addition, China's influence on the global stage and leadership role in the sustainable development agenda provide new perspectives for the study of global environmental governance and international cooperation^11^.

In fact, as an important strategic region in western China, the Chengdu–Chongqing economic Circle has exceeded the national average in terms of economic development and urbanization rate, but problems such as inter-city resource competition, unsustainable land use and deteriorating environmental conditions have seriously hindered the progress of new-type urbanization and ecological welfare performance in this region^12,13^. Therefore, in order to effectively promote the coordinated development of new-type urbanization and ecological welfare performance in Chengdu–Chongqing economic circle and similar areas, it is necessary to deeply study the intricate relationship between these aspects and explore the mechanisms and ways conducive to the mutual promotion of the two.

Presently, scholars have conducted more extensive research on ecological welfare performance and new urbanization. With regard to ecological welfare performance, Zhu^14^ defined ecological welfare performance based on Daly’s ideas^15,16^, providing a foundational framework for understanding the intertwined dynamics between ecological welfare and development. Initially, scholars measured ecological welfare performance using the ratio method, such as by employing ratios like the happy life index to the ecological footprint or the human development index to the ecological footprint^17,18^. Then, methodologies like stochastic frontier analysis (SFA) and data envelopment analysis (DEA) have been proposed, rooted in input–output theories, to measure ecological welfare performance. For instance, He et al. utilized the SFA model to assess ecological welfare performance in Jiangsu Province, China, revealing significant spatial disparities between its northern and southern regions^19^. Similarly, employing the Super-SBM model, Yao et al. evaluated ecological welfare performance across Chinese provinces and municipalities, emphasizing the necessity to enhance resource use efficiency and environmental regulation^20^. Ecological welfare performance is influenced by various factors including urbanization^21^, industrial structure^22^, technological innovation^23^, and green finance^24^. Among these, urbanization stands out as a primary determinant shaping ecological welfare performance. Bao et al. noted an inverted U-shaped relationship between urbanization levels and ecological welfare performance^25^. Different types of urbanization exhibit varied impacts: while population and land urbanization hinder ecological welfare performance enhancement, economic and social urbanization demonstrate positive effects. With regard to new urbanization, departing from traditional urbanization paradigms. The traditional urbanization is only measured by the proportion of the urban population to the total population, which seems to be one-sided and fails to reflect the diversity of modern urban development^26,27^. Simultaneously, it is essential to recognize that China’s unique socio-economic context poses distinct challenges to the conventional urbanization paradigm^28,29^. With its vast population, constrained urban space, and a historical legacy deeply rooted in agriculture, the traditional approach to urbanization may not adequately encapsulate the complexities and nuances of China's urbanization trajectory. This underscores the need for a tailored urbanization strategy that aligns with China's specific conditions and leverages its unique strengths. Therefore, the new urbanization emphasizes the transformation from "population urbanization" to "people-centered urbanization", with the cultivation of residents' sense of gain and happiness as the core of construction^30,31^. This novel approach encompasses five key dimensions: population, economy, society, environment, and space. New urbanization seeks to optimize economic structures, ensure equitable access to public resources, and protect ecological environments while enhancing residents' quality of life^32–34^. Rapid population influx during the new urbanization process imposes significant pressures on the ecological environment, accentuating ecological resource shortages^35,36^. While economic development enhances basic public service provisions and residents' objective welfare levels, challenges like urban sprawl and environmental pollution threaten urban livability, thereby hindering urban ecological welfare performance^37^. Although existing research provides some reference value for this paper, there remains a noticeable gap in understanding their interconnected and coordinated relationship. Investigating this interconnectedness offers valuable insights into urban ecological resource consumption and urbanization trends, guiding the formulation of sustainable urban development strategies.

**Figure 1:**
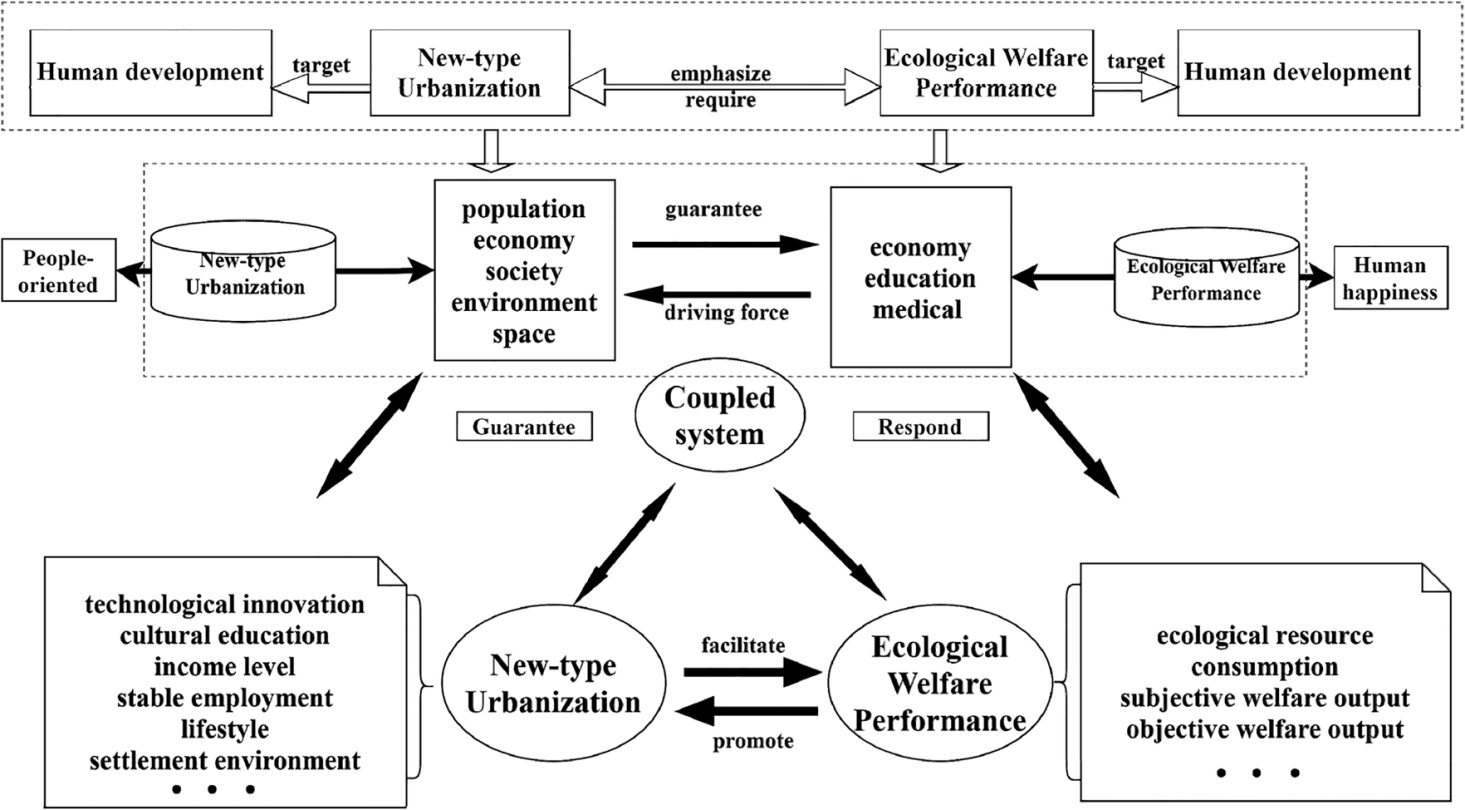
Mechanism of coupling ecological welfare performance and new urbanization.

The study's contributions to the existing literature are manifold. Our study introduces a groundbreaking, holistic framework that connects the realms of ecological welfare and contemporary urban development—a field that has been largely unexplored in academic research (Fig. 1). By applying the GTWR model, we have conducted an in-depth analysis that elucidates the complex dynamics and temporal-spatial fluctuations in the interplay between urban advancement and ecological health. This cutting-edge method surpasses conventional analytical approaches, delivering a more refined and comprehensive understanding of the urbanization-ecological nexus, thereby addressing a significant gap in scholarly knowledge.

Furthermore, the insights from our research significantly enrich the discourse on sustainable urban development and offer pragmatic guidance to urban planners and policymakers. They are now better equipped to achieve a balance between urban expansion and environmental stewardship. The transformative potential of our work is particularly evident in its implications for urban planning strategies within the CCEC and similar urban conglomerates, steering the way towards sustainable, people-centric urban settings.

## Materials and methods

### Study areas and data sources

**Figure 2:**
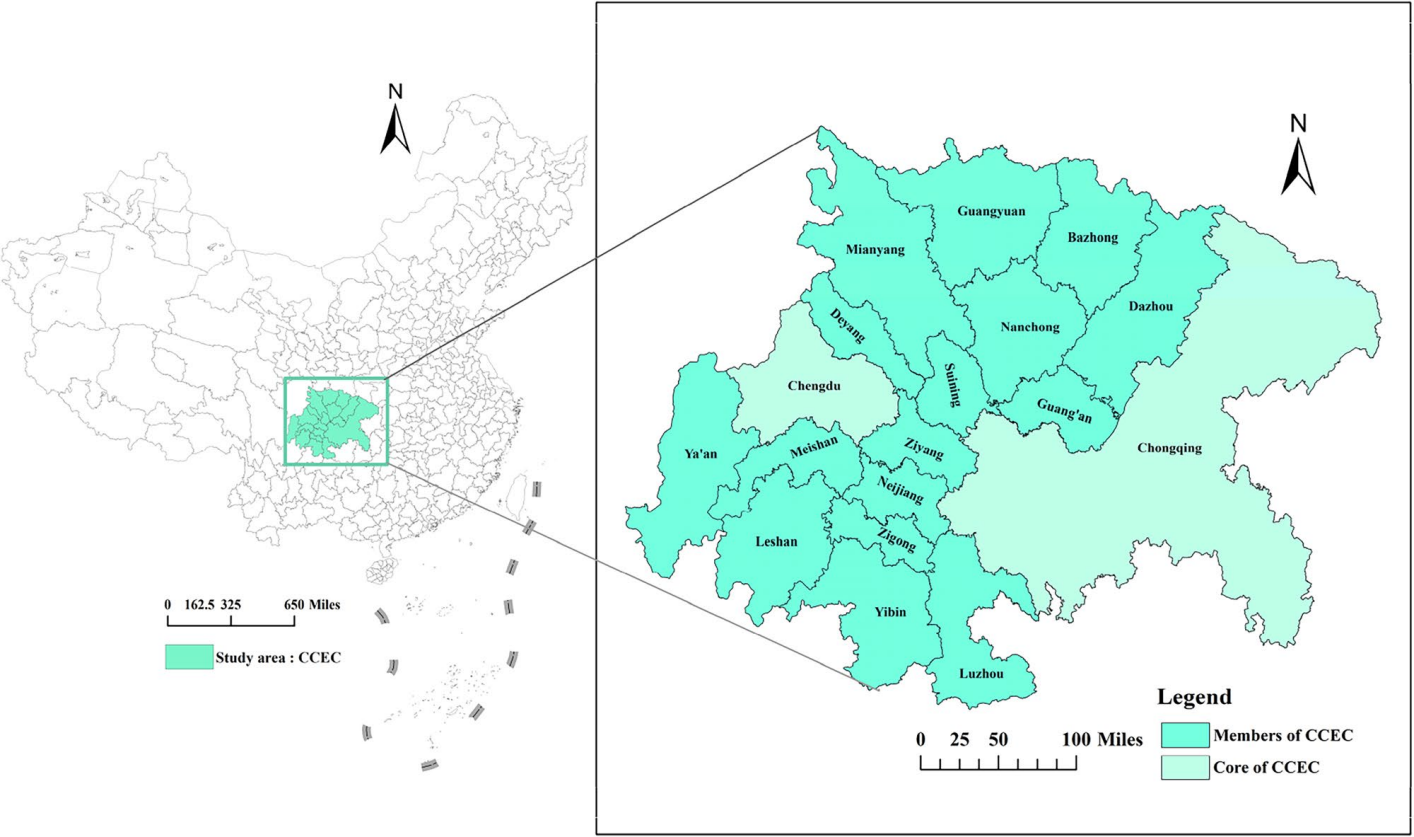
Location of the study area (mapping based on the ArcGIS10.8 sofware can be obtained from the following link, https://desktop.arcgis.com).

The Chengdu–Chongqing Economic Circle (CCEC) is situated in western China, as depicted in Fig. 2. Boasting exceptional ecological endowments, abundant natural resources, and a high concentration of cities and towns, this region stands out as the most densely populated, industrially advanced, innovative, market-oriented, and open area in western China. Its unique and strategically significant position plays a crucial role in the broader context of national development.

Considering that the 10-year timeframe spanning from 2011 to 2020 aligns with the implementation phase of China's 12th and 13th Five-Year Plans, this paper selects the period from 2010 to 2020 to align theoretical research with actual development, aiming to draw more practical conclusions. The research's practical significance lies in its ability to provide valuable insights. The required data for this paper comprises three sections: one for measuring ecological welfare performance, another for evaluating the level of new urbanization, and the third for exploring the influencing factors of the coupling degree of coordination. The pertinent data is sourced from the statistical yearbooks of each city from 2011 to 2021, with any missing data filled in through linear interpolation.

### Research methods

**Figure 3:**
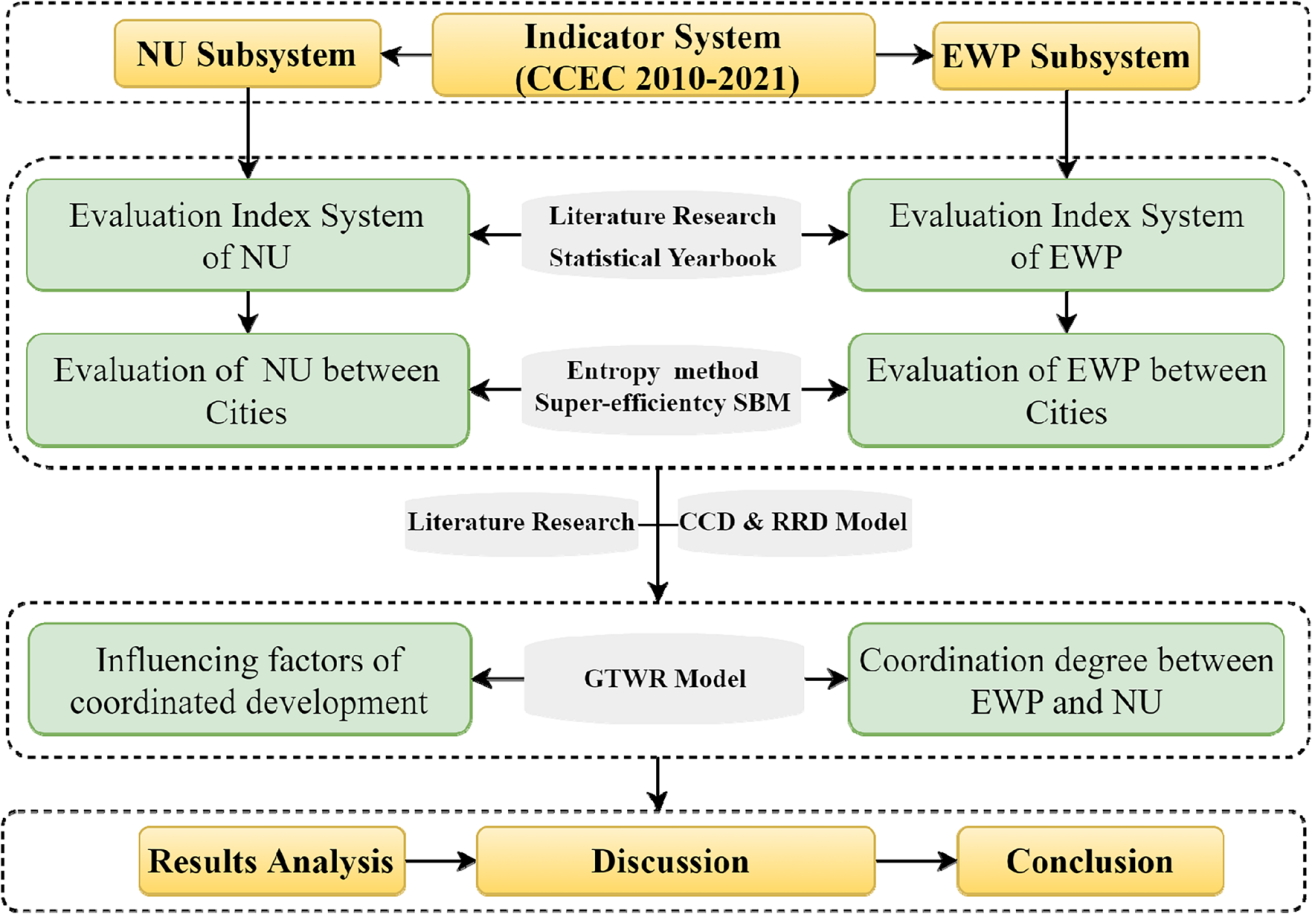
Research framework.

In this study, we reasonably measured and evaluated the coupling coordination measurement and temporal and spatial differences between EWP and NU in the CCEC through the following methods and steps: (1) through literature research and the statistical yearbook, EWP and NU index system in the study area was constructed, and the SBM model and entropy weight were used to calculate the benefits of the index system. (2) CCD was used to analyze the EWP and NU in the CCEC. (3) The temporal and spatial heterogeneity of EWP and NU in the study area was evaluated based on the GTWR model. The research methods and main contents are shown in Fig. 3.

#### Calculated the ecological welfare performance based on the super-efficiency SBM model

##### The measurement index system of ecological welfare performance

The ecological welfare performance reflects the efficiency of transforming ecological resources into human welfare under solid sustainability. Based on the input–output model, an urban ecological welfare performance evaluation index system is established (Table 1). Both expected and non-expected output are considered; non-expected output is represented by environmental pollution, and the social welfare level represents desired output.

**Table 1:** The evaluation indicator system of ecological welfare performance.

Category	Primary index	Secondary index	Three-level index
Input	Resource consumption	Land	Per capita urban construction land area
		Energy	Per capita household electricity consumption
		Water	Per capita water supply
Output	Expected	Solid waste discharge	Per capita production of municipal solid waste
			Per capita production of general industrial solid waste
		Wastewater discharge	Per capita industrial wastewater discharge
			Chemical oxygen demand emissions per capita
		Exhaust emission	Per capita sulfur dioxide emission
			Per capita smoke (dust) emissions
	Non-expected	Economics	Per capita GDP
			Total social retail goods per capita
			Per capita fiscal revenew urbanizatione
		Education	Average years of education
		Medical treatment	Amount of doctors per 10,000 people
			Amount of hospital beds per 10,000 people
			Amount of employees insured by basic medical insurance

##### The super-efficiency SBM model

As a relaxed DEA model, the SBM solves the problem that the input and output variables can only change in equal proportion in the radial model^38^. The super efficiency model overcomes the problem that the efficiency values of DMU cannot be further compared^39^. The super-efficiency SBM model combines the advantages of the two models, relaxes the input and output variables, and distinguishes the effective DMU to more accurately measure the relative relationship between the efficiency values of cities in the study area^40,41^. At the same time, in measuring ecological welfare performance, the input variable is the primary variable of the decision-making unit and the influencing factor that causes the change in output results. Therefore, this study adopts an input-oriented super-efficiency SBM model to measure the urban ecological welfare performance.

Under variable returns to scale, there are DMUs, denoted as $DMU_{j}$($j = 1,2, \ldots ,n$); Each DMU has $m$ inputs $x_{i}$ ($i = 1,2, \cdots ,m$) and $q$ species produce $y_{r}$ ($r = 1,2, \cdots ,q$). The super-efficient SBM model is expressed as follows:

1 $$ \begin{aligned} & \min \rho = 1 + \frac{1}{m}\sum\limits_{i = 1}^{m} {\frac{{s_{i}^{ - } }}{{x_{ik} }}} \\ & s.t.\left\{ {\begin{array}{*{20}c} {\sum\limits_{j = 1,j \ne k}^{n} {x_{ij} \lambda_{j} - s_{i}^{ - } \le x_{ik} } } \\ {\sum\limits_{j = 1,j \ne k}^{n} {y_{rj} \lambda_{j} + s_{i}^{ + } \ge y_{rk} } } \\ {\lambda ,s^{ - } ,s^{ + } \ge 0} \\ {i = 1,2, \ldots ,m;\;r = 1,2, \ldots ,q;\;j = 1,2, \ldots ,n;\;(j \ne k)} \\ \end{array} } \right. \\ \end{aligned} $$

where, the $\rho$ is the ecological welfare performance of each city, and the larger the value, the higher the ecological welfare performance; $x_{ik} ,y_{rk}$ are the input and output variable values of the k city, respectively; The vectors are relaxation variables of input and output, respectively; $\lambda$ is the weight vector.

#### Evaluation index system of new urbanization

##### Data standardization

In order to eliminate the differences in the dimensions of each index, formulae (2) and (3) are used for data standardization:

Positve:

2 $$ Z_{ij}^{ + } = \frac{{X_{ij} - \min (X_{j} )}}{{\max (X_{j} ) - \min (X_{j} )}} + 0.001 $$

Negative:

3 $$ Z_{ij}^{ - } = \frac{{\max (X_{j} ) - X_{ij} }}{{\max (X_{j} ) - \min (X_{j} )}} + 0.001 $$

where, the $Z$ represents standardized data, the $X_{ij}$ represents the original data of the *j*th indicator in the *i*th year, $\max (X_{j} )$ and $\min (X_{j} )$ represent the *j*th indicator’s maximum and minimum values, respectively.

##### Entropy method

The entropy method is an objective weighting technique that calculates indicator weights by assessing the degree of dispersion in the indicator data^42^. Given the substantial regional variations in basic conditions, regional characteristics, and development trends, this study employs the global entropy method to analyze the evaluation indices of new urbanization both vertically and horizontally. This approach also helps mitigate the subjective influence of human factors to a certain extent^43^.

The calculation formula are as follows:

4 $$ p_{ij} = \frac{{Z_{ij} }}{{\sum {Z_{ij} } }} $$

5 $$ e_{j} = - \frac{{\sum {p_{ij} \cdot \ln (p_{ij} )} }}{\ln (m)} $$

6 $$ \omega_{j} = 1 - \frac{{e_{j} }}{{\sum {(1 - e_{j} )} }} $$

7 $$ NU_{i} = \sum {Z_{ij}^{ + } (Z_{ij}^{ - } )} \cdot \omega_{j} $$

where, the $NU_{i}$ represents new urbanization evaluation value, the $\omega_{j}$ represents entropy weight.

Adhering to the principles of scientific rigor and representativeness, and drawing from existing research, this study selected 13 indicators across five dimensions—population, economy, society, environment, and space—to construct the evaluation index system for new urbanization. The weights of these indicators were determined using the entropy value method (see Table 2).

**Table 2:** New urbanization evaluation index system.

Target	Primary Index	Secondary indicators & attributes	Weight	Unit	References
New urbanization	Population urbanization	Proportion of urban population (+)	0.0842	%	^44, 45^
		Urban population density (+)	0.0930	Persons/km^2^	
		Registered urban unemployment rate (−)	0.0390	%	
	Economic urbanization	Per capita GDP (+)	0.1263	RMB	^46, 47^
		Per capita urban disposable income (+)	0.1476	RMB	
	Social urbanization	Enrolment in higher education per 100,000 population (+)	0.0803	Person	^48, 49^
		Number of beds in health facilities per 10,000 population (+)	0.0180	Bed	
		Public transportation vehicles per 10,000 population (+)	0.0698	Vehicle	
	Environmental urbanization	Non-hazardous domestic waste disposal rate (+)	0.0232	%	^50^
		Area of green space per capita (+)	0.1081	m^2^/person	
		Greening coverage of built-up areas (+)	0.0330	%	
	Spatial urbanization	Built-up area as a proportion of urban area (+)	0.1060	%	^44^
		Urban road area per capita (+)	0.0717	m^2^/person	

#### Coupling coordination degree model

The coupling coordination degree model is frequently employed to analyze the degree of coordination between systems. Its measurement primarily encompasses calculating the coupling degree, coordination index, and coupling coordination degree of three indicators. Eventually, by combining the coupling coordination degree value with the classification criteria for coordination, the system's degree of coordination is determined^51^. The formula is as follows:

8 $$ C = 2\left[ {\frac{{EWP_{ij} \cdot NU_{ij} }}{{(EWP_{ij} + NU_{ij} )^{2} }}} \right]^{1/2} $$

9 $$ T = \gamma \times EWP_{ij} + \varepsilon \times NU_{ij} $$

10 $$ D = \sqrt {C \cdot T} $$

where, the $EWP$ represents the normalized ecological welfare performance value, the $C$ represents the degree of coupling between the ecological welfare performance of the j-th city and the level of new urbanization in year i, taking a value between 0 and 1. A higher value indicates a stronger coupling between the two systems. The $T$ represents the comprehensive coordination index of the two systems, where $\gamma$ and $\varepsilon$ are coefficients to be determined. Given that both systems hold equal importance in urban development, we set $\gamma = \varepsilon = 0.5$. The $D$ denotes the coupling coordination degree of the two systems, with a value ranging from 0 to 1. A higher value indicates a stronger coupling coordination degree between the two systems.

#### Relative degree of development model

While the CCDM proficiently assesses the level of coupled and coordinated development between ecological welfare performance and new urbanization, it lacks the ability to reflect the relative development of the two. Hence, Formula (11) is employed to calculate the relative development index of the two systems, providing a measure that reflects the relative degree of development between them.

11 $$ RDD = \frac{{EWP_{ij} }}{{NU_{ij} }} $$

where, the $RDD$ represents the relative development degree between EWP and NU. Based on relevant research^52^, this paper categorizes the coupling status of ecological welfare performance and new urbanization into five levels and three types (see Table 3).

**Table 3:** Ecological welfare performance and new urbanization coupling coordination type classification.

Types of coordinated development	degree of coupling coordination	Grade	Relative degree of development	Types of relative development
Coordinated development	(0.8, 1.0]	Highly coordination	RD > 1	New urbanization lagging
			RD ≈ 1	Synchronization
			RD < 1	Ecological welfare performance lagging
	(0.6, 0.8]	Moderate coordination	RD > 1	New urbanization lagging
			RD ≈ 1	Synchronization
			RD < 1	Ecological welfare performance lagging
Transitional development	(0.4, 0.6]	Basic coordination	RD > 1	New urbanization lagging
			RD ≈ 1	Synchronization
			RD < 1	Ecological welfare performance lagging
Disordered development	(0.2, 0.4]	Moderate uncoordination	RD > 1	New urbanization lagging
			RD ≈ 1	Synchronization
			RD < 1	Ecological welfare performance lagging
	(0, 0.2]	Highly uncoordination	RD > 1	New urbanization lagging
			RD ≈ 1	Synchronization
			RD < 1	Ecological welfare performance lagging

#### GTWR model

Geographically weighted regression (GWR) models can address the limitations of traditional econometric models that neglect spatial effects, assume homogeneity among variables a priori, and derive coefficients based solely on regional averages^53^. The analysis of influencing factors on urban ecological welfare performance and the synergistic development of new urbanization should acknowledge the presence of spatial non-stationarity and heterogeneity. While the GWR model is limited in observing changes at different time points, the GTWR model incorporates the time dimension into regression parameters. This allows for local regression analysis for each observation unit at various time points, offering a more comprehensive reflection of the magnitude and direction of indicator impact at different geographic locations and times^54,55^. The equation for the GTWR model is:

12 $$ Y_{i} = \alpha_{0} (u_{i} ,v_{i} ,t_{i} ) + \sum\limits_{k = 1}^{n} {\alpha_{k} (u_{i} ,v_{i} ,t_{i} )x_{ik} + \theta_{i} } $$

In this equation, $Y_{i}$ represents the coupling coordination degree of City i, and $x_{ik}$ stands for the k-th influencing factor of the synergistic development of City i's ecological welfare performance and new urbanization.$(u_{i} ,v_{i} ,t_{i} )$ represents the spatio-temporal coordinates of the i-th city.$\alpha_{0} (u_{i} ,v_{i} ,t_{i} )$ is a constant;$\alpha_{k} (u_{i} ,v_{i} ,t_{i} )$ is the regression coefficient of the k-th influence factor; $\theta_{i}$ is the random error term.

Parameter estimation was conducted through locally weighted least squares, calculated as:

13 $$ \widehat{\alpha }(u_{i} ,v_{i} ,t_{i} ) = [{\mathbf{X}}^{{\mathbf{T}}} {\mathbf{W}}(u_{i} ,v_{i} ,t_{i} ){\mathbf{X}}]^{ - 1} \times {\mathbf{X}}^{{\mathbf{T}}} {\mathbf{W}}(u_{i} ,v_{i} ,t_{i} ){\mathbf{Y}} $$

In this context, $\widehat{\alpha }(u_{i} ,v_{i} ,t_{i} )$ represents the parameter to be estimated in the GTWR model, while ${\mathbf{W}}(u_{i} ,v_{i} ,t_{i} )$ is the spatio-temporal weight matrix.

## Results and analysis

### Spatio-temporal evolution characteristics of EWP and NU

#### Temporal trend

**Figure 4:**
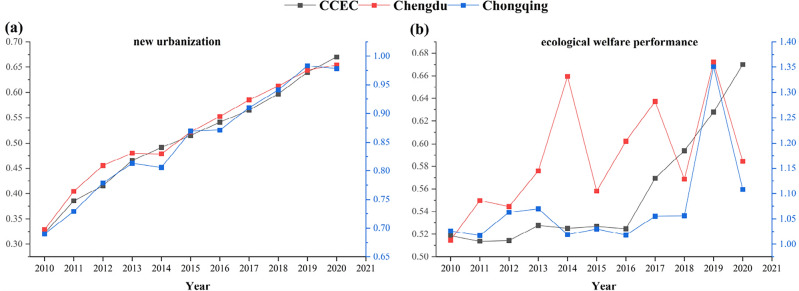
Development trend of ecological welfare performance and new urbanization in the Chengdu–Chongqing Economic Circle from 2010 to 2020.

The ecological welfare performance (EWP) and new urbanization (NU) values for each city in the economic circle from 2010 to 2020 were determined using the super-efficiency SBM model (Eq. 1) and the entropy weight comprehensive evaluation method (Eqs. 2–7). The average values of the 18 cities for each year are presented in Fig. 4. From 2010 to 2020, both ecological welfare performance and new urbanization show an increasing trend. The level of ecological welfare performance increased from 0.519 to 0.670, with an average annual increase of 2.59%; the level of new urbanization increased from 0.322 to 0.670, with an average annual increase of 7.6%.

#### Spatial characteristic

**Figure 5:**
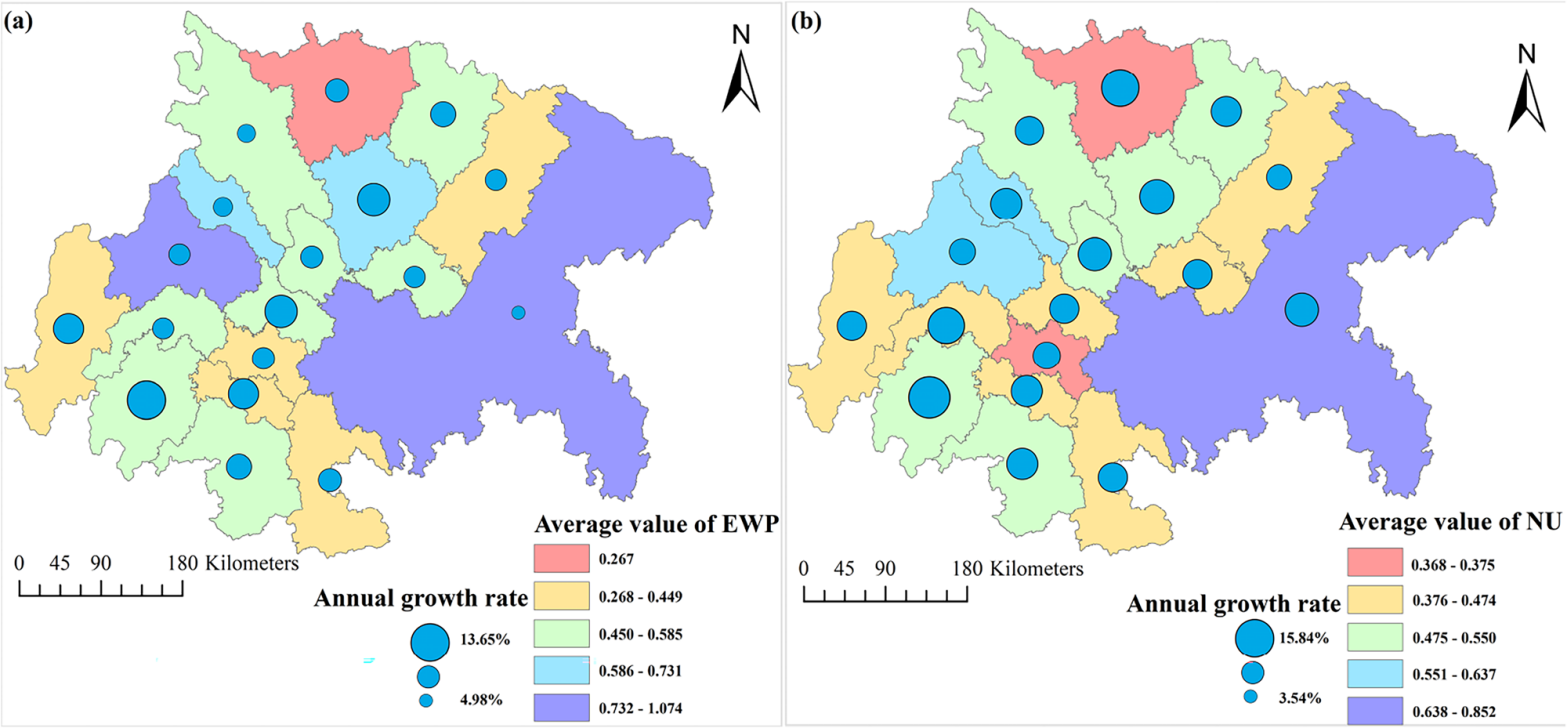
Spatial distribution characteristics of ecological welfare performance and new urbanization in the Chengdu–Chongqing Economic Circle from 2010 to 2020 (Mapping based on the ArcGIS10.8 sofware can be obtained from the following link, https://desktop.arcgis.com).

Utilizing ArcGIS10.8 software to visualize the ecological welfare performance and new urbanization of each city in the economic circle over the years, Fig. 5 is generated. The level of ecological welfare performance in each city is generally on an upward trend, with individual cities (Deyang, Nanchong and Guang'an) showing a "downward trend in the early period and a rebound in the later period". The difference in the average level of ecological welfare performance among different cities is obvious, and there is an imbalance, but the degree of imbalance is weakening over time. There is also a clear trend of growth in the level of new urbanization in each city, with the highest average level of new urbanization in the core cities of Chengdu and Chongqing, reaching 0.852 and 0.637 respectively.

### Spatio-temporal evolution characteristics of CCD

#### Temporal trend of CCD

**Figure 6:**
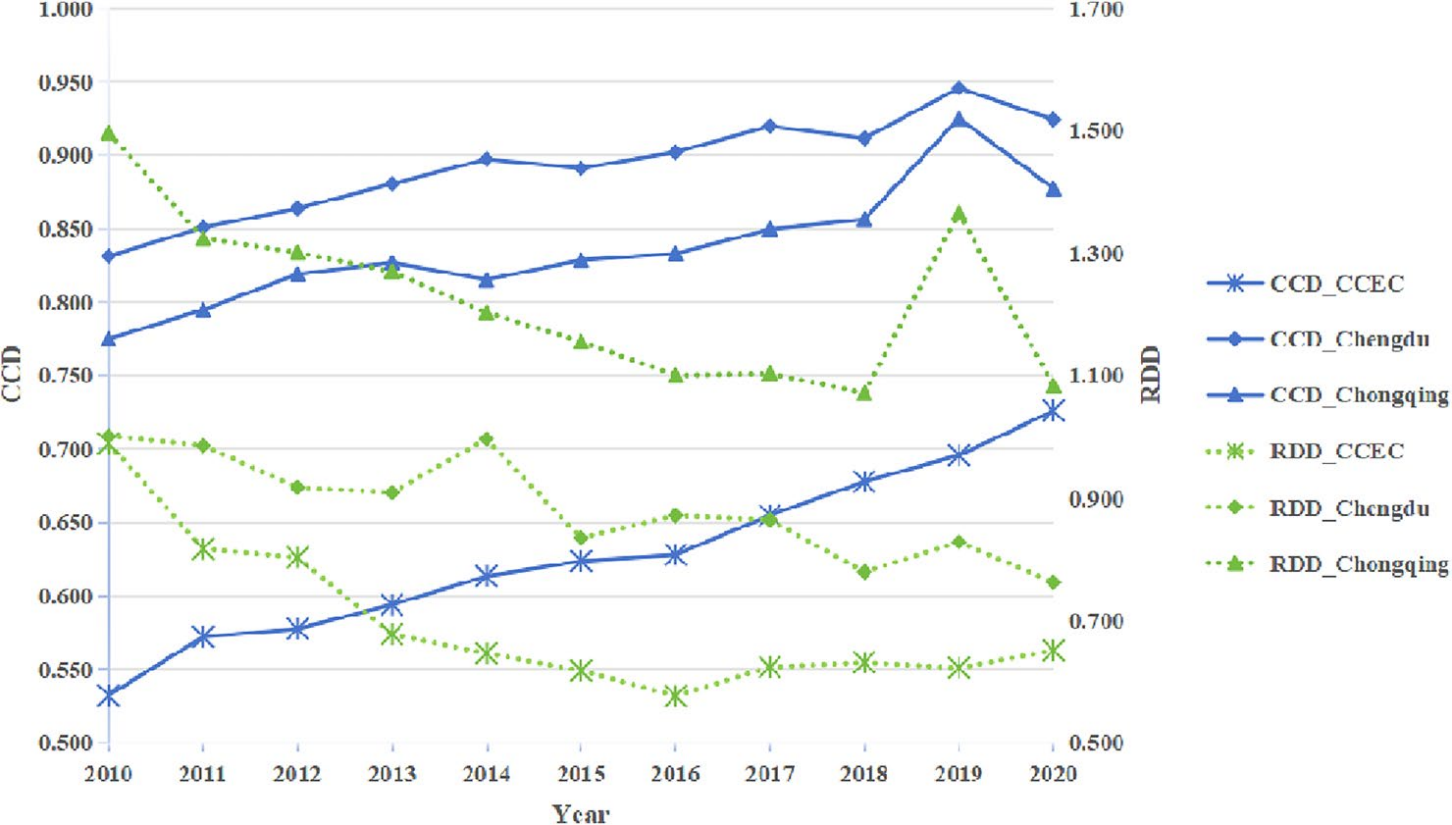
The coupling and coordinated development trend of ecological welfare performance and new urbanization in the Chengdu–Chongqing Economic Circle from 2010 to 2020.

According to the coupling coordination degree model and the relative development degree model discussed in the previous section, the coupling coordination degree and relative development index of the 18 cities in the economic circle from 2010 to 2020 were calculated. The average values for each year were then determined to characterize the ecological welfare performance of the economic circle, the coupling coordinated development level of new urbanization, and the relative development level of the Chengdu–Chongqing Economic Circle for the respective year, as illustrated in Fig. 6.

As depicted in Fig. 6, on one hand, the coupling coordination degree of ecological welfare performance and new urbanization in the Chengdu–Chongqing Economic Circle exhibits a substantial growth trend from 2010 to 2020, rising from 0.532 in 2010 to 0.726 in 2020. Concerning the coordination level, the average coupling coordination degree of the economic circle during the period 2010–2013 is below 0.6, indicating a transitional stage of basic coordination. From 2014 to 2020, the average coupling coordination degree of the economic circle falls within the range of moderate coordination. Notably, the coupling degree of Chengdu and Chongqing, the two core cities of the Chengdu–Chongqing Economic Circle, consistently surpasses the average level, reaching a highly coordinated level.

On the other hand, the average value of the relative development index for the economic circle's ecological welfare performance and new urbanization demonstrates a decreasing trend and consistently remains below 1. This suggests that the CEEC as a whole is in a developmental stage characterized by lagging ecological welfare performance. Chengdu shares a similar development type, albeit with a lower degree of lag, while Chongqing is in a lagging state of new urbanization development. Nevertheless, Chongqing's trajectory indicates a decreasing trend, with ecological welfare performance and new urbanization gradually converging toward synchronous development.

#### Spatial characteristic of CCD

**Figure 7:**
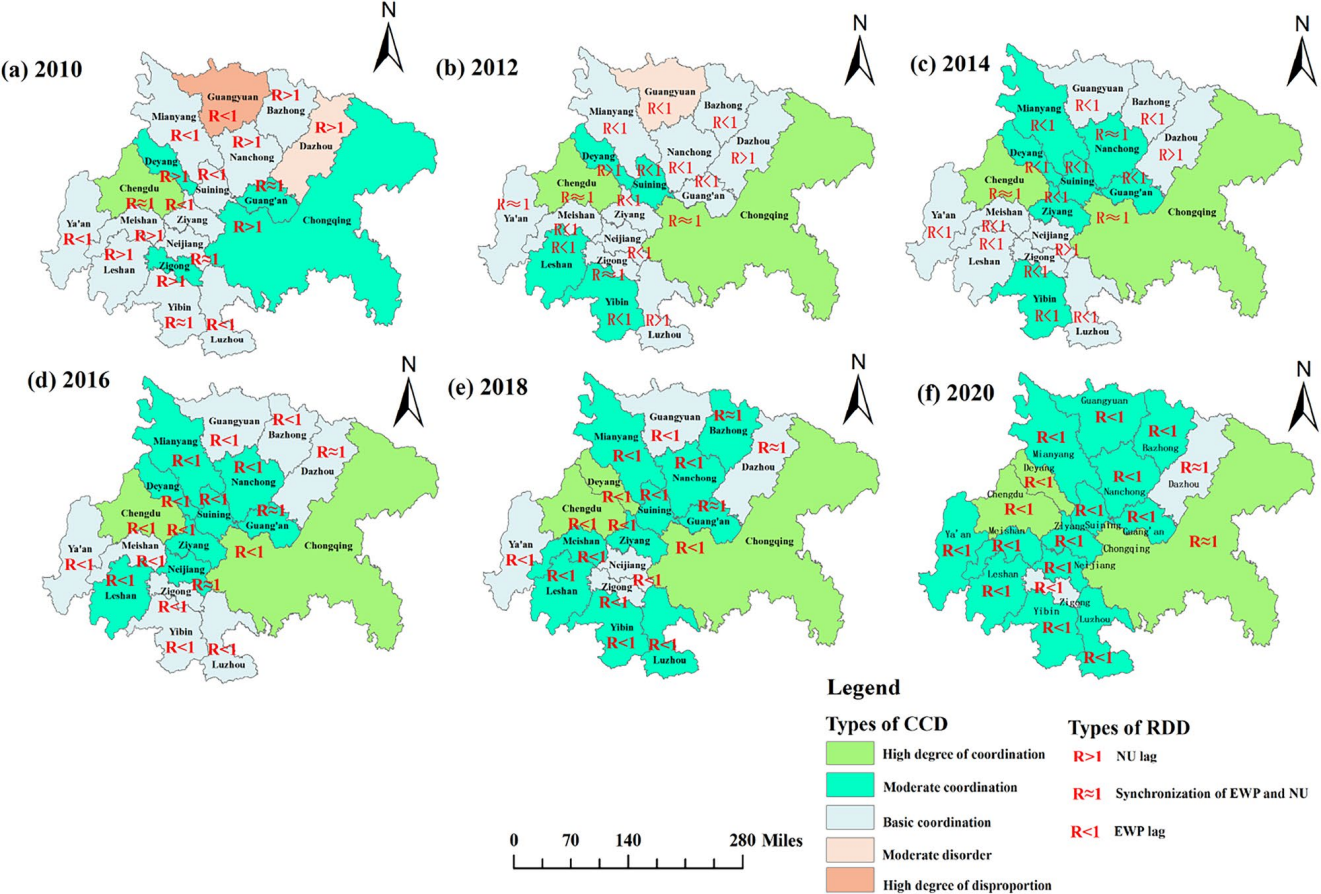
Spatial distribution characteristics of the CCD & RDD in the Chengdu–Chongqing Economic Circle from 2010 to 2020 (Mapping based on the ArcGIS10.8 sofware can be obtained from the following link, https://desktop.arcgis.com).

In order to examine the structural changes in the coupled coordination of ecological welfare performance and new urbanization in the economic circle, this paper calculates the number of cities of each coordination type in each year and visualizes the spatial distribution, as shown in Fig. 7. As can be seen from Fig. 7, the structure of the coupled coordination between the ecological welfare performance of the economic circle and new urbanization has been optimized continuously, with the number of cities of low coordination types decreasing and the number of cities of high coordination types expanding. Overall, it is divided into two stages, with the basic coordination type dominating in 2010–2014, and evolving into the medium coordination type dominating in 2016–2020 driven by the two core cities of Chengdu and Chongqing.

### Analysis of influencing factors of CCD

#### Variable selection

In this study, the method described in Sect. 2.2.3 is employed to gauge the degree of coordination in the coupling of ecological welfare performance and new urbanization. Four indicators are chosen to investigate the influencing factors of coupling and coordinated development, considering both internal and external aspects in alignment with existing studies. Among these, city scale: *City_S*, the number of urban population, the higher the proportion of urban population, the larger the city^22^; Industrial structure upgrading: *Ind*, the level of advanced industrial structure, the higher the proportion of tertiary industry indicates the industrial structure^23^; Economic development: *Econ*, per capita GDP, the level of economic development is represented by per capita GDP after adjustment; Green technology innovation: *Gtec*, the number of authorized green invention patents, the higher the proportion of green invention patents, the higher the green technology innovation ability^11^.

#### Data processing and model fitting

Considering the variations in magnitude among influencing factors, standardization was employed to normalize the data. The GTWR model was estimated using ArcGIS 10.2 software with the GTWR plug-in toolkit, opting for automatic optimization to set the bandwidth and configuring the spatio-temporal distance parameter ratio to 1. Simultaneously, to assess the applicability of the GTWR model, the data underwent OLS regression using Stata 15.1 software. GWR and GTWR were also applied to the data using ArcGIS 10.2 software, and the performance parameters of the models are presented in Table 4.

**Table 4:** GTWR model parameters.

Model	OLS	GWR	GTWR
Bandwidth		0.111	0.114
Sigma		0.045	0.038
AICc	− 442.397	− 537.086	− 464.96
RSS	1.180	0.409	0.281
*R*^2^	0.646	0.878	0.916
Adjusted *R*^2^	0.637	0.876	0.914

#### Time series analysis of influencing factors

**Figure 8:**
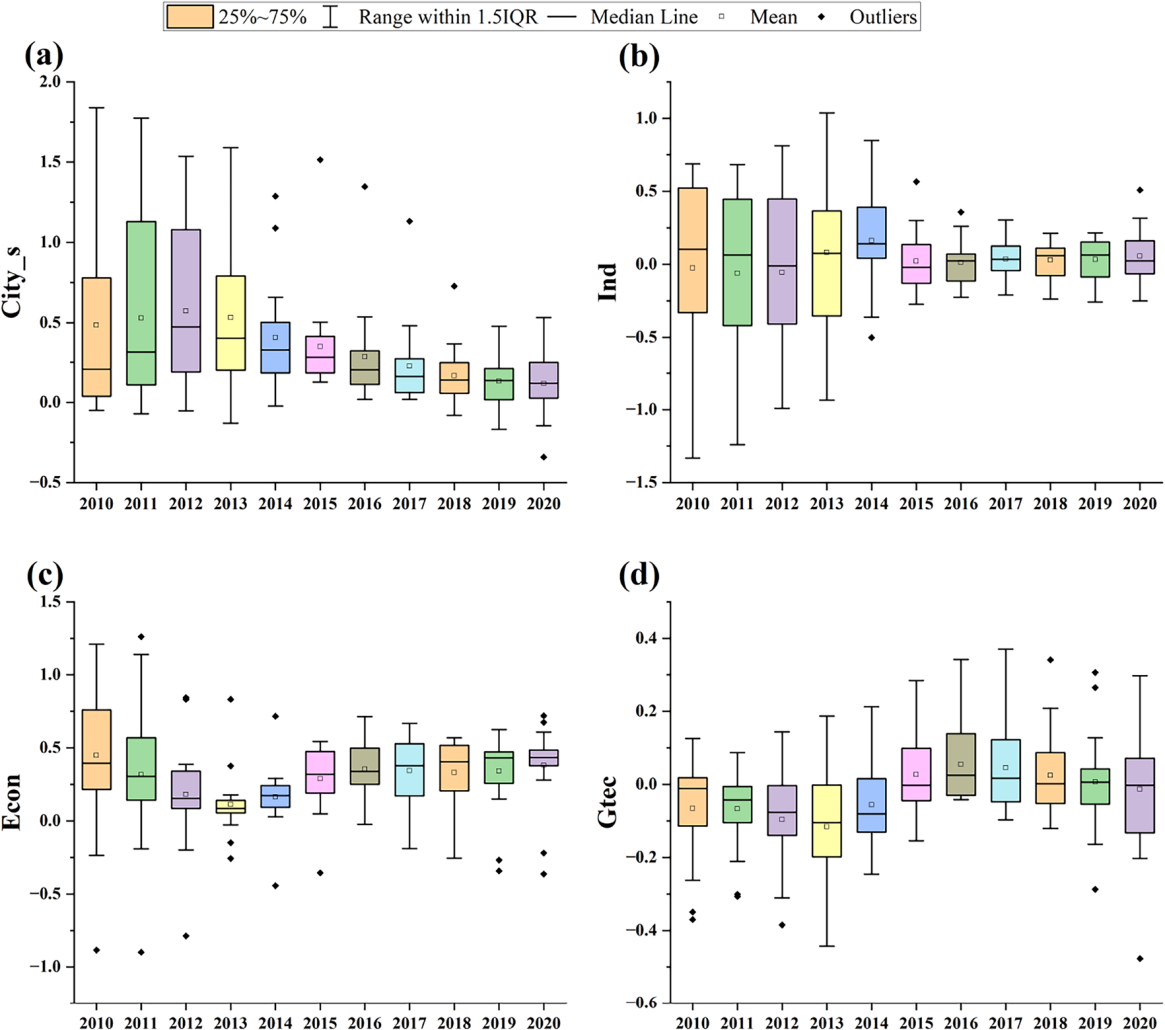
Time-series variation of regression coefficients of the GTWR model.

The analysis conducted spatio-temporal geographically weighted regression on the factors influencing the coupling and coordination of ecological welfare performance and new urbanization in the economic zone from 2010 to 2020. The relationship of the coefficients over time was visualized, as illustrated in Fig. 8, to observe the trend of temporal evolution.

The impact of city size (Fig. 8a) on the coupling of ecological welfare performance and new urbanization in the economic zone shows a positively decreasing trend, accompanied by a reduction in the degree of dispersion. The influence of advanced industrial structure (Fig. 8b) on the coupling and coordination of ecological welfare performance and new urbanization in the economic circle exhibits both positive and negative effects, but the overall impact is not substantial, lying within the range of [− 0.5, 0.5]. The level of economic development (Fig. 8c) has the most significant impact on the coupling and coordination of ecological welfare performance and new urbanization in the economic area, presenting a positive "V"-shaped trend of decreasing and then increasing, with a sharp increase after 2013. It indicates that since 2013, the CCEC has experienced a pronounced V-shaped upward trajectory in economic impact, a trend intimately connected to the 2011 rollout of the Regional Plan for the Chengdu–Chongqing Economic Zone. This strategic initiative has served as a catalyst for transformation within the economic circle by delivering a refreshed development blueprint and robust policy support. It has facilitated a pivotal shift from conventional manufacturing to cutting-edge industries and services, while concurrently intensifying the focus on ecological civilization and environmental conservation throughout the urbanization process. As the plan's execution has progressed, the CCEC has become a magnet for substantial investment, particularly in sectors of green technology and innovation. These investments have progressively yielded returns, acting as a significant impetus for the economy's swift ascent. Consequently, the post-2013 economic resurgence delineated by a V-shaped pattern underscores the affirmative outcomes of economic restructuring and industrial evolution within the CCEC, steered by national policy directives. It signifies the nascent yet promising synchronization between economic prosperity and ecological welfare performance as the region advances in new urbanization endeavors. The impact of green technology innovation (Fig. 8d) on the coupling and coordination level of ecological welfare performance and new urbanization in the economic circle exhibits a negative decreasing trend, and the impact is relatively small, with the mean value of the impact coefficient falling within the range of [− 0.1, 0.1].

#### Spatial analysis of influencing factors

**Figure 9:**
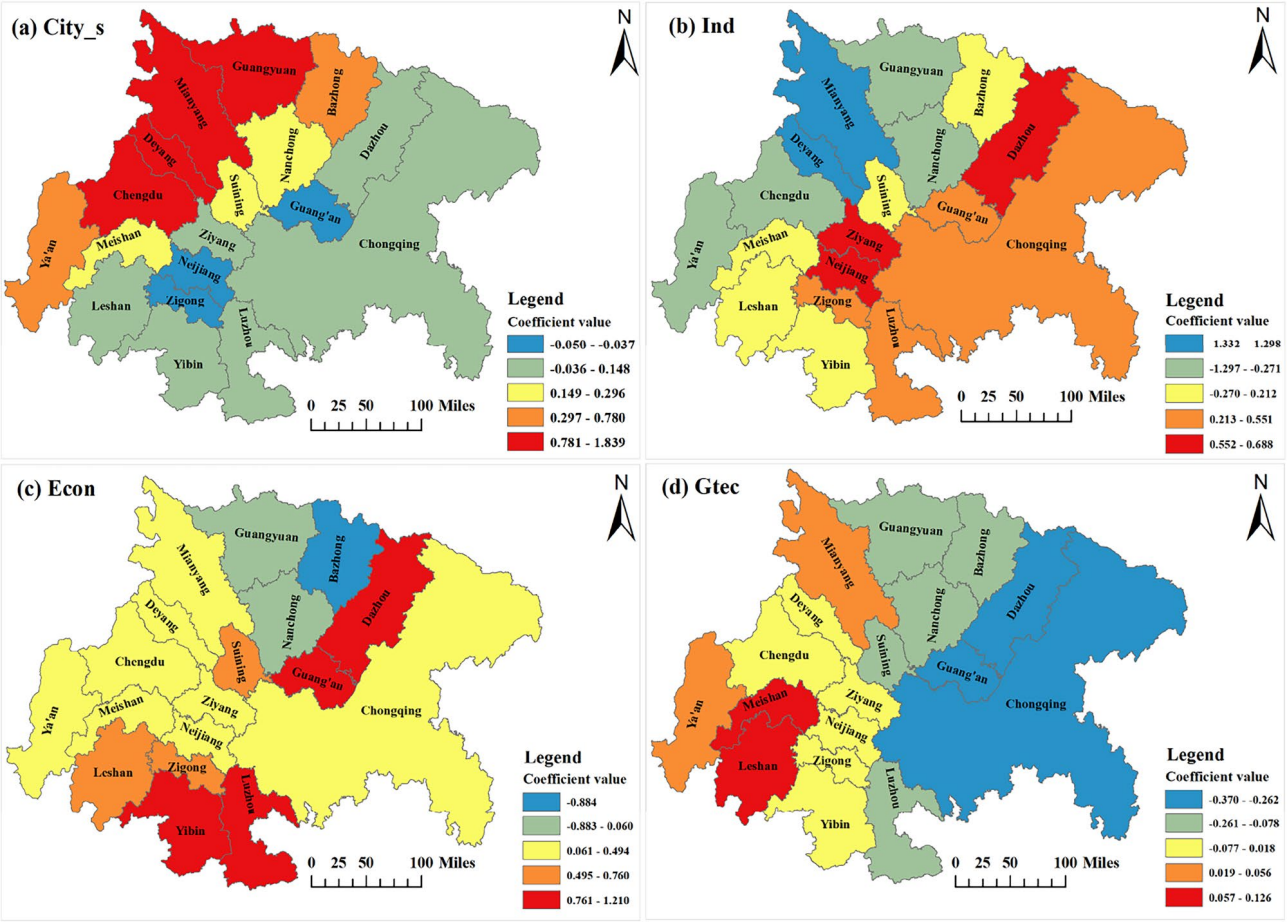
Spatial variation in regression coefficients of the GTWR model (Mapping based on the ArcGIS10.8 sofware can be obtained from the following link, https://desktop.arcgis.com).

Leveraging ArcGIS10.8, the mean values of the GTWR regression coefficients for each year were visualized and presented spatially. This visualization aimed to analyze the influencing factors of the coupled and coordinated development of the ecological welfare performance of the economic circle and the new urbanization, culminating in Fig. 9.

The regression coefficients of city size (Fig. 9a) are prominently negative only for Guang’an, Neijiang, and Zigong, with a relatively low degree of influence. The regression coefficients for advanced industrial structure (Fig. 9b) roughly follow a pattern of "Chongqing and its radiating cities > Chengdu and its radiating cities". In the regression coefficients for the level of economic development (Fig. 9c), only three cities—Guangyuan, Bazhong, and Nanchong—show negative effects, with Bazhong exhibiting the largest negative effect. The spatial distribution of the regression coefficient for green technological innovation (Fig. 9d) displays evident polarization, with Chengdu at the core of the economic circle region mainly showing a positive effect, while Chongqing at the core of the economic circle region is dominated by a negative effect.

## Discussions

### Establishment of a coupled coordination framework

Previous studies have often focused on singular aspects of either ecological welfare performance or new urbanization, overlooking their intrinsic interrelation^56–58^. To address this gap, our study introduces a coupled coordination framework (Fig. 1) that underscores their interconnectedness. This framework aims to foster a symbiotic relationship between urban development and ecological welfare, facilitating a win–win scenario. Firstly, by managing the relationship between urban development and ecological welfare, the framework seeks to mitigate irreversible ecological damage caused by urbanization^59–61^. Secondly, by considering ecological welfare performance comprehensively, we aim to enhance cities’ resilience to environmental challenges, thereby promoting sustainable development^62–64^. Lastly, integrating ecological welfare performance with new urbanization enables a more holistic assessment of urban development quality, ultimately enhancing residents' quality of life^65,66^.

### Measures for coordinated development of EWP and NU

The empirical findings regarding the factors influencing the degree of coupled coordination reflect the current state of ecological welfare performance and new urbanization within the Chengdu–Chongqing Economic Circle. To enhance the positive interaction between the ecological environment and urbanization, it is imperative to propose measures at both macro and micro levels. These measures will not only elevate the Chengdu–Chongqing Economic Circle but also offer valuable insights and strategies for the development of other regions. The subsequent sections delve into a detailed discussion of these potential measures.

#### Macro level

Our findings indicate an imbalance in the ecological welfare performance within the Chengdu–Chongqing Economic Circle, as well as regional disparities in the level of new urbanization, alongside a relatively lower degree of coupled and coordinated development (Figs. 5, 6, 7). At the macro level, it is essential for the core cities of Chengdu and Chongqing to intensify their efforts in ecological resource management, environmental protection, and enhancing the well-being of their residents. This proactive approach will significantly augment the radiation-driven capacity of these two cities^67–69^. Then, given the shift from lagging urbanization to lagging ecological and environmental welfare performance within the Chengdu–Chongqing Economic Circle, it is imperative for government authorities and administrators to recognize that hasty urbanization has led to detrimental effects such as surface vegetation depletion, environmental pollution, and disruption of ecosystem equilibrium^70–72^. Therefore, embracing a steadfast commitment to ecological prioritization and green development must serve as the cornerstone of urbanization initiatives. This entails continually optimizing the allocation of ecological resources throughout the urbanization process and fostering the effective integration of urbanization with the ecological environment^73^.

#### Micro level

The GTWR model unveils the spatial and temporal variations in the impact of factors on ecological welfare performance and the coordinated development of new urbanization (Figs. 8, 9). This serves as a critical foundation for contemplating the synchronized advancement of regional ecology and environment at the micro level. Firstly, this study measures city size by total urban population, and empirical results confirm that China’s "demographic dividend" initially fostered the synergistic development of urbanization and the environment^74^. However, as urban population expansion remains unchecked, this facilitative effect has transformed into a hindrance. Hence, it is imperative for the government to optimize the urban population structure, expedite the construction of eco-cities, and refine urban spatial arrangements. Secondly, owing to the implementation of China's high-quality economic development strategy, the impact of economic growth exhibits a "V" shape^75,76^. Inadequate investment in green technology exacerbates the adverse trajectory of its impact on coupling coordination^77,78^. Consequently, it is advisable for the government to bolster financial investment in environmental governance and steer enterprises towards intensifying research and development in green technology. This entails providing targeted R&D support for eco-friendly solutions such as polluted wastewater treatment and renewable energy^79^.

#### Community, education and participation: drivers of coupling and coordination

Navickienė’s research highlights the key role of sustainable communities, education and stakeholder engagement in promoting socially and environmentally compatible development^80^. Therefore, in addition to the above macro and micro level measures, the importance of sustainable communities, education, and stakeholder engagement in promoting the coordinated development of NU and EWP should be emphasized^81,82^. First, sustainable communities are now recognized as incubators of green lifestyles, contributing to the EWP by raising residents' environmental awareness and promoting green lifestyles^83^. Secondly, sustainable education plays an important role in cultivating citizens' environmental awareness and stimulating innovative thinking^84^, which will be conducive to the improvement of green technology innovation ability and thus enhance the efficiency of ecological resource utilization^85^. Finally, establish strong stakeholder engagement mechanisms to ensure that policy and planning initiatives are aligned with community needs and values^86^, thereby facilitating coordination between NU and EWP.

### Limitations and future research directions

This study offers an exhaustive appraisal of the interplay between NU and EWP, underpinned by a rigorous and deliberate selection of pertinent indicators. Our analysis is further enriched through the application of the CCDM and GTRW, which facilitate a nuanced exploration of the subject matter. Despite the thoroughness of our approach, there are aspects of the research that necessitate further investigation. Notably, the correlation between the multifaceted dimensions of NU and their EWP is an area earmarked for future research, with the grey relational model positioned as a pivotal analytical tool in this endeavor. Moreover, acknowledging the projected regional variations in the levels of coupling and coordination, we propose to incorporate quantile regression models. This methodological choice aims to meticulously assess the impact of various factors across a spectrum of coupling and coordination intensities, thereby enhancing our understanding of the complex dynamics at play.

## Conclusions

This study examines the intricate relationship between ecological welfare performance (EWP) and new urbanization (NU) within the Chengdu–Chongqing economic circle from 2010 to 2020. In this research, we initially observed a robust positive trend between EWP and NU, indicating a synergistic and harmoniously coordinated progression. Nonetheless, the decelerating growth rate of EWP in comparison to NU underscores an exigent requirement for urban administrators to implement strategies that augment ecological resource stewardship. Specifically, enhancing the efficacious utilization of critical resources such as water and land is paramount to elevating the comprehensive welfare of urban inhabitants, thereby fortifying EWP. Subsequently, the substantial impetus provided by the metropolitan hubs of Chengdu and Chongqing has catalyzed a notable upsurge in the coupling coordination levels of NU and EWP. By the year 2020, an impressive majority—over 88.9% of the cities—had attained a moderate state of coupling coordination between the two indices. This trend reflects a collective and affirmative movement towards integrated development across the region. Consequently, it is imperative for policymakers within the Chengdu–Chongqing economic sphere to seize this propitious juncture and devise policies aimed at fostering equilibrium in the development trajectory of NU and EWP. Ultimately, the impact of diverse factors on the coupled and coordinated development of EWP and NU manifests pronounced spatiotemporal variations. Notably, the economic development's V-shaped rebound pattern corroborates the efficacy of the 2016 strategic integration of Chengdu and Chongqing. Concurrently, the variegated influence of green technological innovation impels urban planners to prioritize urban demographic governance, ecological resource optimization, and investment in research and development. These elements are identified as pivotal in escalating the coordinated development index of EWP and NU.

Our research underscores the importance of refining urban governance, ecological resource allocation, and spatial planning, with green technology innovation being pivotal for sustainable urban growth. These insights inform policy initiatives aimed at aligning urbanization with ecological welfare enhancement. While our study contributes to the discourse on sustainable urban development, it recognizes its limitations in capturing the full spectrum of NU and its interplay with EWP. Future studies should explore the grey correlation model to deepen the understanding of the complex interaction between NU and EWP, and at the same time explore the complex performance of various influencing factors at different urbanization levels through quantile regression model, so as to provide new ideas for promoting the coordinated development strategy of EWP and NU and promoting urban sustainable development.

## Data Availability

The data used in this study can be provided by the corresponding author upon request.
